# Evaluating hop extract concentrations found in commercial beer to inhibit *Streptococcus mutans* biofilm formation

**DOI:** 10.1111/jam.15632

**Published:** 2022-06-05

**Authors:** Eric R. Gregory, Renad F. Bakhaider, Grace F. Gomez, Ruijie Huang, Elizabeth A. S. Moser, Richard L. Gregory

**Affiliations:** ^1^ Department of Pharmacy Services The University of Kansas Health System Kansas City Kansas USA; ^2^ Department of Biomedical Sciences and Comprehensive Care Indiana University School of Dentistry Indianapolis Indiana USA; ^3^ Department of Biostatistics Indiana University School of Medicine Indianapolis Indiana USA

**Keywords:** biofilms, dental caries, hops, *Streptococcus mutans*, α‐acids, β‐acids

## Abstract

**Aims:**

The purpose of this study was to compare the effect of hop extracts with diverse β‐acid concentrations on *Streptococcus mutans* biofilm formation.

**Methods and Results:**

Ten different hop extracts, with α‐acid concentrations similar to those found in commercial beer products and β‐acid concentrations ranging from 2.6 to 8.1%, were added to distilled water to make standardized concentrations. *S. mutans* isolates were treated with hop extract dilutions varying from 1:2 to 1:256. The minimum inhibitory, minimum bactericidal and minimum biofilm inhibitory concentrations were determined and the optical density was evaluated. Live/dead staining confirmed the bactericidal effects. Biofilm formation of several strains of *S. mutans* was significantly inhibited by hop extract dilutions of 1:2, 1:4, 1:8, 1:16 and 1:32. Strong negative correlations were observed between α‐ and β‐acid concentrations of the hop extracts and *S. mutans* total growth and biofilm formation.

**Conclusions:**

The use of hop extracts prepared similarly to commercial beer decreased *S. mutans* biofilm formation.

**Significance and Impact of the Study:**

The inclusion of hops in the commercial beer products may provide beneficial health effects. Further studies are warranted to determine an effect in vivo on the development of dental caries.

## INTRODUCTION

Various plant compounds have been used for millennia to aid humans in their quest to eradicate disease (Tronina et al., 2020; Palombo, 2011). The female flower of *Humulus lupulus*, commonly known as the hop plant, has been utilized for numerous purposes over the centuries, indicated for infection, cancer, atherosclerosis, diuresis, pain, inflammation and sleep disorders (Lin et al., 2019). Additionally, the plant was added to commercial goods hundreds of years ago for its valuable preservative properties (Hrnčič et al., 2019). While the specific product to which it was first added is controversial, its most widely known application was to avoid spoilage of beer during global voyages from England to India (Tronina et al., 2020).

Polyphenols are one group of the numerous chemicals found in hops which stand out. Included in the polyphenol group is the α‐acid, or humulone, which gives the distinctive bitter taste, and the β‐acid, also known as lupulone. Researchers have extracted these polyphenols and tested their potential for human use. Unique to the β‐acid, antimicrobial activity has been one of the most widely described properties (Cermak et al., 2017; Gerhauser, 2005; Kramer et al., 2014; Shinada et al., 2007; Yaegaki et al., 2008). These β‐acids inhibit the growth of many infection‐causing Gram‐positive genera, such as *Bacillus*, *Micrococcus*, *Staphylococcus*, *Streptococcus* and *Streptomycetes* (Gerhauser, 2005). Of these, *Streptococcus mutans* has been implicated as a primary aetiological pathogen in dental caries along with *Lactobacilli* spp., *Actinomyces* spp. and *Veillonella* spp.

*S. mutans*, in particular, forms an oral biofilm utilizing extracellular polysaccharide on the affected tooth to promote colonization (Yaegaki et al., 2008). The mechanism by which this occurs has been described as the initial electrostatic binding of *S. mutans* to salivary pellicle glycoproteins. This is followed by bacterial receptor–salivary pellicle ligand interactions including *S. mutans* antigen I/II binding to salivary agglutinin increasing the strength of the binding to the tooth surface. Once oral bacteria attach to the tooth surface, they thrive and form biofilms. *S. mutans*‐containing biofilm metabolizes sucrose in the oral cavity with lactic acid as a major end product of glycolysis, leading to tooth decay (Dashper & Reynolds, 1996). On the other hand, caries are easily preventable with adequate oral hygiene and avoidance of fermentable carbohydrates (Chapple et al., 2017). While brushing and flossing can cause transient bacteraemia, colonization of organisms such as *S. mutans* coupled with mucosal or dental insults can lead to systemic infections. Persistent bacteraemia and, eventually, infective endocarditis are possible complications due to chronically poor dentition (Baddour et al., 2015).

In addition to optimal dental care, another strategy for the prevention of dental caries could be utilization of the antibacterial effects of β‐acids in hops. Although not commonly found in medication, numerous types of hops are available commercially; however, the β‐acid concentrations fluctuate between products. Therefore, various hops may differentially inhibit overall *S. mutans* growth and oral biofilm formation. While β‐acids have been studied in detail, the question remains if various whole hop extracts can reduce in vitro biofilm formation of *S. mutans*. The purpose of this study was to prepare hop extract dilutions of varying β‐acid concentrations in a similar manner as commercial beer products and assess the ability of the preparations to inhibit *S. mutans* total growth and biofilm formation in vitro.

## MATERIALS AND METHODS

### Bacterial strains, media and growth conditions

*S. mutans* strains UA159 (ATCC 700610), UA130, OMZ‐175, LM7, A32‐2, NG8 and 10,449 (Nassar et al., 2012; Nassar & Gregory, 2017) were used in the present study and stored at ‐80°C in tryptic soy broth (TSB, Acumedia, Baltimore, MA) containing 20% glycerol before use. Mitis salivarius sucrose bacitracin (MSSB, Anaerobe Systems, Morgan Hill, CA) agar plates were used to initially grow the organisms. Unless otherwise stated, TSB was used and the bacteria were grown in 5% CO_2_ at 37°C.

### Preparation of hop extracts

The 10 different hop extracts included in the study are noted in Table 1. The hops (Hopunion LLC, Yakima, WA) were purchased in vacuum‐sealed pouches from a local brewing supply store. α‐ and β‐acid compositions were obtained from the manufacturer. Upon purchase, the dried hop extracts were added to distilled water to create a standardized 6.6 mg/ml for all 10 extracts. These mixtures were placed in a hot water bath and boiled for 90 min, then filter sterilized and diluted in TSB to concentrations ranging from 1:2 to 1:256 to imitate concentrations commonly found in commercially available beer.

**TABLE 1:** Alpha‐ and β‐acid concentrations of hop extracts

Hop extract	Initial α‐acid concentration (percent)	Initial β‐acid concentration (percent)	Quantity of β‐acid used in biofilm assay (mg)
Styrian Celeia	3.2	2.6	0.172
Liberty	4.5	3.5	0.231
German Hallertau	2.7	3.8	0.251
Nugget	13.5	4.5	0.297
Czech Saaz	3.6	4.8	0.317
Cluster	8.1	5.2	0.343
German Northern Brewer	9.6	5.5	0.363
Amarillo	8.2	6.2	0.409
Cascade	7.8	6.6	0.436
Glacier	5.0	8.1	0.535

### Determination of MIC and MBC

The minimum inhibitory concentration (MIC) and the minimum bactericidal concentration (MBC) of each hop extract for the *S. mutans* isolates were determined using a twofold dilution method as described earlier by this laboratory (Huang et al., 2012). Briefly, a single colony of *S. mutans* from an MSSB plate was inoculated into 5 ml of TSB and incubated overnight. Ten microlitre of the overnight culture of *S. mutans* (approximately 10^6^ colony‐forming units [CFU] determined by spiral plating) in TSB was treated with 190 μl of 1:2–1:256 dilutions of each of the hop extracts in quadruplicate in TSB supplemented with 1% sucrose (TSBS) for 24 h in sterile 96‐well flat‐bottom microtitre plates (Fisher Scientific, Newark, DE, USA). The optical density (OD) values of the total growth of the bacterial cultures were measured at 595 nanometres in a spectrophotometer (SpectraMax 190; Molecular Devices, Sunnyvale, CA, USA). The MIC was defined as the lowest concentration of the hop extracts that yielded a significantly decreased change in OD compared to the 0 hop control. Bacterial cultures (10 μl) from the wells with hop extract dilutions of 1:2, 1:4, 1:8 and 1:16 were transferred onto blood agar plates (Fisher Scientific, Newark, DE, USA) and incubated for 48 h to assess the MBC. The MBC was defined as the lowest concentration of the hop extracts that had no visible bacterial colonies on agar plates after 48 h of incubation.

### Determination of biofilm formation and MBIC

The minimum biofilm inhibitory concentration (MBIC), defined as the lowest concentration of an agent that significantly inhibits the visible biofilm formation of a micro‐organism, was determined following the measurement of the total growth absorbance as optimized previously (Huang et al., 2012). The culture supernatant was removed, the biofilm was gently washed four times with saline, then fixed with 200 μl of 10% formaldehyde, washed and stained with 200 μl of 0.5% crystal violet for 30 min (Huang et al., 2012). After washing the stained biofilm, crystal violet was extracted from the biofilm cells by incubation for 1 h with 200 μl of 2‐propanol. The extract was diluted 1:5 with 2‐propanol and the absorbance was read at 490 nm with 2‐propanol used as the blank control.

### Ability of hop extracts to inhibit established biofilm of other *S. mutans* strains and microscopic analysis

A pool of the hop extract was prepared by mixing equal volumes of each of the different hops. The pooled hop extract was diluted in TSBS and added (200 μl) to 24‐hour‐old established *S. mutans* biofilms from the seven strains in 96‐well microtitre plates. The pooled hop extract dilutions were incubated for another 24 h on the established biofilms and stained with crystal violet as described above. In order to visually confirm the MBC results, live/dead staining of *S. mutans* strain UA159 was conducted by mixing 5 μl of the hop‐treated cultures from the microtitre plates with live/dead *Bac*Light bacterial viability stain (ThermoFisher Scientific) for 10 min and examined under a fluorescent microscope (Model number BZ‐X810; Keyence Corporation of America, Itasca, IL) at 1000X.

### Statistical analysis

Each experiment was conducted in triplicate. The results are presented as mean and standard error of mean. SPSS Statistics version 16 (SPSS Inc., Chicago, IL) was used for data analysis. Due to non‐normality, a rank transformation was used prior to one‐way ANOVA. The one‐way ANOVA and the post hoc Tukey multiple comparisons test were performed to compare the means of the hops extract treated and untreated groups. A significance level of α = 0.05 was adopted for statistical hypothesis testing. Spearman correlations were used to determine the statistical significance of correlations between the α‐ and β‐acid content with total growth and biofilm formation and with each other.

## RESULTS

### Effect of hop extracts on growth and biofilm formation

**FIGURE 1:**
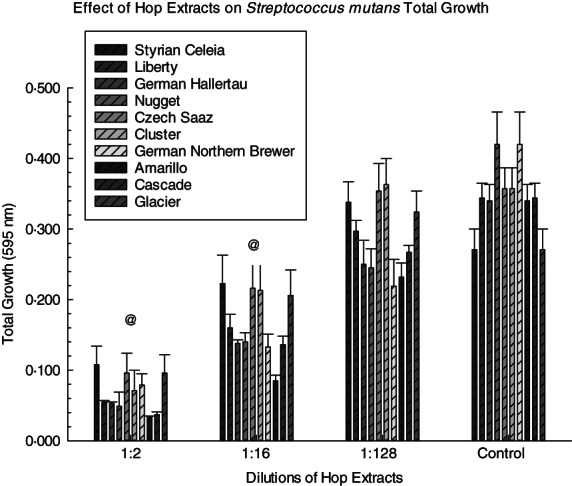
Effect of hop extracts on *S. mutans* UA159 total growth. *S. mutans* was treated with 1:2, 1:16 and 1:128 dilutions of each extract for 24 h. Significant differences in individual hop extracts at the 1:128 dilution compared to the 0 hop extract control groups were demonstrated for the Styrian Celeia, German Hallertau, Nugget, German Northern Brewer, Amarillo and Cascade hop extracts. @ symbols indicate that all the hop extracts at that dilution (1:2 and 1:16) were significantly different from the 0 hop extract controls.

**FIGURE 2:**
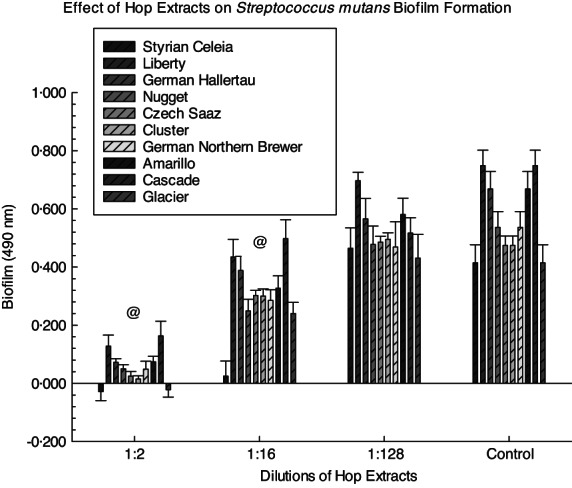
Effect of hop extracts on initial *S. mutans* UA159 biofilm formation. *S. mutans* was treated with 1:2, 1:16 and 1:128 dilutions of each extract for 24 h. Significant differences in individual hop extracts at the 1:128 dilution compared to the 0 hop extract control groups were demonstrated for the Cascade hop extract. @ symbols indicate that all the hop extracts at that dilution (1:2 and 1:16) were significantly different from the 0 hop extract controls.

Significant inhibition of *S. mutans* UA159 total growth and biofilm formation was noted with the addition of each hop extract compared to the controls (Figures 1 and 2, respectively). The hop extracts had significant ability to inhibit the growth of *S. mutans* UA159 as evidenced by the MIC for each extract (Table 2). This was observed for each hop extract in the 1:2 (*p* < 0.01), 1:4 (*p* < 0.01), 1:8 (*p* < 0.01), 1:16 (*p* < 0.01) and 1:32 (*p* = 0.05) dilutions. In addition, the MIC values for the pooled hops extract ranged from 1:8 to 1:64 with the exception of *S. mutans* NG‐8 that demonstrated an MIC with the pooled hops extract of <1:4. Two of the hop extracts (German Northern Brewer and Amarillo) displayed significant inhibition of biofilm formation at a 1:256 dilution (*p* < 0.01 and *p* = 0.02, respectively; Tables S1 and S2).

**TABLE 2:** Minimum inhibitory concentrations (MIC) of various hop extracts on *S. mutans* UA159

	Hop Extracts MICs^a^									
*S. mutans* strain	Styrian Celeia	Liberty	German Hallertau	Nugget	Czech Saaz	Cluster	German Northern Brewer	Amarillo	Cascade	Glacier
UA159	1:2	1:16	1:128	1:128	1:16	1:16	1:128	1:128	1:16	1:16

^a^ MIC is represented as a dilution of the hop extract that inhibits growth. Higher MIC dilutions reflect stronger inhibition of growth.

### Correlation of the effect of hop extracts on growth and biofilm formation

Strong negative correlations were demonstrated between the inhibition caused by the hop extracts and their α‐acid and β‐acid content as the *S. mutans* total growth (−0.701, *p* < 0.0001; −0.690, *p* < 0.01) and biofilm formation (−0.611, *p* < 0.0001; −0.663, *p* < 0.01), respectively, decreased in the presence of the hop mixtures. The 1:2, 1:4 and 1:8 dilutions of each hop extract demonstrated no growth in the MBC analysis while the 1:16 dilutions exhibited growth. Therefore, an MBC of 1:8 for all hops extracts was measured. The α‐ and β‐acid compositions of the 10 hop extracts were significantly correlated with total growth and biofilm formation (Table 3). Furthermore, the amount of α‐acids and β‐acids were not strongly correlated with each other, but the correlation was statistically significant (Spearman correlation of 0.27666; *p* < 0.0001).

**TABLE 3:** Correlation of total growth and biofilm growth with α‐ and β‐acid composition of the 10 hop extracts

	α‐acids		β‐acids	
Inhibition assay	Spearman correlation	*p*‐value	Spearman correlation	*p*‐value
Total growth	−0.70113	<0.0001	−0.69027	<0.0001
Biofilm	−0.61094	<0.0001	−0.66260	<0.0001

### Effect of hop extracts on established biofilm of other *S. mutans* strains and microscopic analysis

**FIGURE 3:**
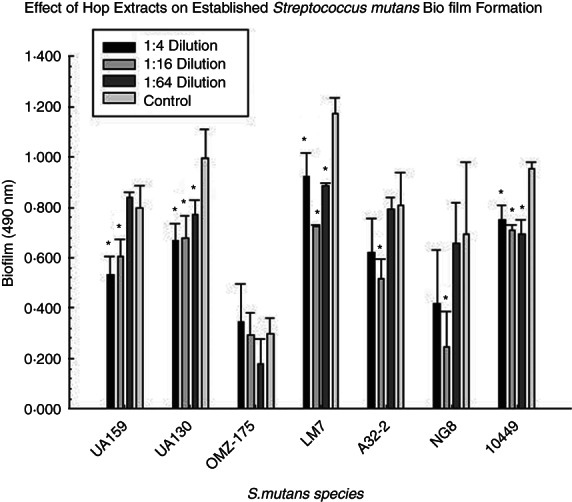
Effect of dilutions of pooled hop extract on established biofilm formation of seven strains of *S. mutans*. Twenty‐four hour established *S. mutans* biofilms were treated with 1:4, 1:16 and 1:64 dilutions of a pool of the hop extracts for another 24 h. Significant differences in the pooled extract on six of the seven *S. mutans* strains were demonstrated. Asterisks indicate that the pooled hop extract at that dilution were significantly different from the 0 hop extract controls.

**FIGURE 4:**
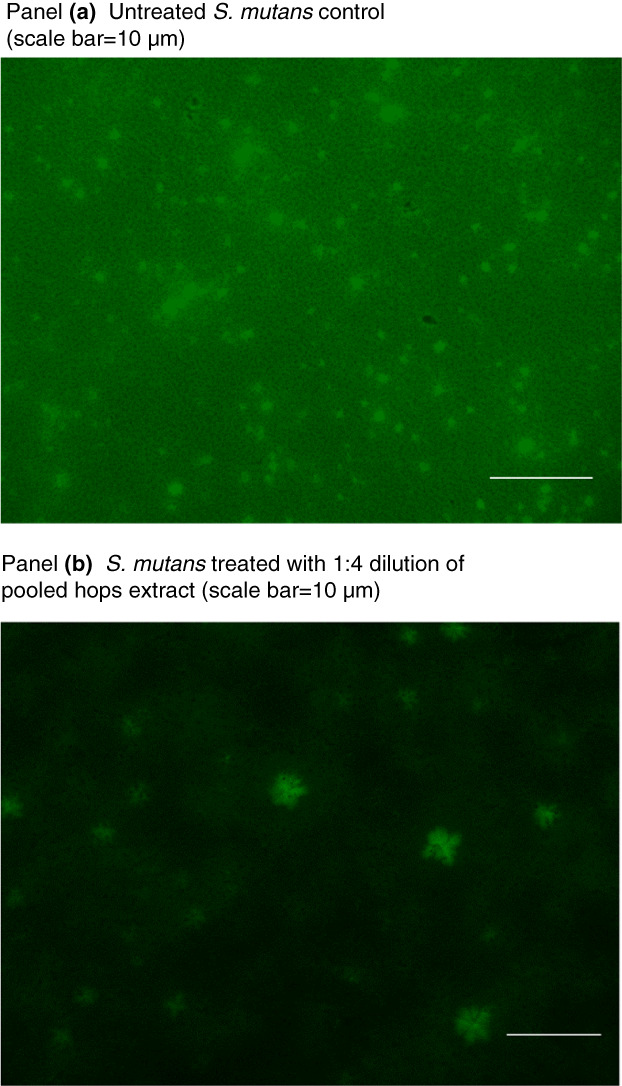
Live/dead staining of *S. mutans* UA159. Panel A: Untreated *S. mutans* UA159 cells. Panel B: Effect of a 1:4 dilution of the pooled hop extract on *S. mutans* UA159 cells.

Each of the seven different *S. mutans* strains produced different levels of biofilm confirming our earlier results (Nassar et al., 2012; Nassar & Gregory, 2017). Significant differences in the pooled hop extract on established biofilms of six of the seven different *S. mutans* strains were demonstrated compared to the 0 hop control values (Figure 3). This indicates that the hop extract provides cross‐strain inhibition of *S. mutans* biofilm and is not just effective against a single strain (*S. mutans* UA159). This was also the case with MIC. In addition, live/dead staining of *S. mutans* UA159 demonstrated killing of the bacterium compared to the 0 hop control (Figure 4).

## DISCUSSION

These data suggest that both α‐ and β‐acids may inhibit total growth and biofilm formation of *S. mutans*. Gram‐positive organisms, such as *S. mutans*, have lipophilic regions of the cell membrane which may serve as a site of antibacterial activity for the β‐acids of hop extracts (Gerhauser, 2005). The mechanism of action of the β‐acids against *S. mutans*, and other Gram‐positive organisms, is thought to be associated with the high pKa of β‐acids, especially when compared to that of α‐acids (Bogdanova et al., 2018; Kramer et al., 2014). With the pKa of 5.5–7.8 for β‐acids compared to 4.0–5.5 for α‐acids, the comparatively increased pKa was found to facilitate the entry of β‐acids through the cell membrane (Steenackers et al., 2015). This may result in a decreased intracellular pH and subsequent interference of essential enzyme reactions, reduced lactic acid production and eventually cell death via cell wall lysis (Kramer et al., 2014; Steenackers et al., 2015).

With an objective of assessing the benefit of diverse hop extracts, the current study examines the ability of the hop extract dilutions to inhibit total growth and biofilm growth of *S. mutans* UA159 compared to a control group in vitro. The strain of *S. mutans* used is the most commonly studied strain and has been evaluated by this lab in comparison to other strains with similar results using nicotine and fluoride, which suggest the effect of hop extracts may be effective against all strains of *S. mutans* (Huang et al., 2012). In this regard, our studies provide evidence for the effect of the hop extracts on at least six strains of *S. mutans*. Our work also indicates the effectiveness of killing provided by the hop extracts. The exposure of the hop extracts to *S. mutans*, combined with the very high negative correlations of the different dilutions and biofilm growth, suggests α‐ and/or β‐acids were likely responsible for the strong inhibition of total growth and biofilm formation. However, since the α‐ and β‐acid compositions are correlated with each other and because previous reports indicate β‐acids are the major antimicrobial in hops, we speculate our results are due to the β‐acid in the 10 hop extracts and not the amount of α‐acids. While the individual hop dilutions varied in α‐ and β‐acid concentrations, each was prepared in a similar manner to how beer is commercially brewed today. Therefore, it is possible that hop extracts prepared by this method may have a role in preventing dental caries when these data are evaluated in humans.

Previous data have shown the benefit of specific hop products in vivo. For example, Yaegaki et al. evaluated if hop polyphenols, when harvested from raw hop plants and administered in salivary‐soluble table form in concentrations of 0.001%, 0.01%, 0.1% and 0.5%, could reduce the growth of *S. mutans* in vitro and in vivo (Yaegaki et al., 2008). In vitro, the 0.5% hop polyphenols inhibited *S. mutans* growth while lactic acid production significantly decreased when exposed to the two most highly concentrated hop polyphenols, 0.1% and 0.5% (Yaegaki et al., 2008). Interestingly, in the randomized single‐blind study of 28 healthy subjects, who received hop polyphenol 20 mg tablets seven times daily, found a lower plaque scoring system compared to the control group. Another double‐blind crossover study found a 0.1% hop polyphenol mouth rinse given five times daily significantly lowered the accumulation of plaque on buccal and lingual teeth surfaces (Shinada et al., 2007). As a result of these studies, one could hypothesize the direct application of hop polyphenols provides antibacterial effects and limits the development of dental caries.

More recently, Leonida et al studied the utility of encapsulating hop extracts in nanochitosan matrices as a means of improving the drug delivery system (Leonida et al., 2018). Chitosan is a polysaccharide which appears to be biocompatible and biodegradable when combined with hop extracts. This polysaccharide has numerous beneficial health effects including antimicrobial, antioxidant and immunomodulating uses (Dai et al., 2011; Kong et al., 2010; Rampino et al., 2013). The incorporation of chitosan into nanoparticles confers even greater benefits such as higher surface: volume ratio, surface charge and biological activity (Zakharova et al., 2015). When combined with β‐acids and xanthohumol, another hop extract, the nanochitosan matrices were found to have broad‐spectrum antimicrobial activity, with inhibitory effects not only against Gram‐positive organisms but also against Gram‐negatives and *Candida* species (Leonida et al., 2018). Therefore, synergy was observed, suggesting nanochitosan matrices may be a worthwhile vehicle for the administration of hop extracts to fight dental caries.

Using a more natural method of drug delivery, the use of whole hop extracts formulated in a similar technique as used in the beer brewing process may provide benefit against organisms causing caries. The value of these preparations was observed in vitro during our study as *S. mutans* biofilm formation was decreased. Moving forward, additional studies are warranted to determine the effect of hop extracts on other common species which cause dental caries. Furthermore, the effect of whole hop extracts on preformed biofilm should be evaluated to assess any potential benefit against pre‐existing plaque, both in vitro and in vivo. If successful, commercial beer may subsequently be assessed for its candidacy to inhibit biofilm formation in vivo.

## CONCLUSION

The results of this study demonstrated the ability of hop extracts to inhibit growth, and initial and established biofilm formation of several different strains of *S. mutans* at concentrations similar to what would be found in beer. Future in vivo investigations are required to confirm the clinical benefits of using hop extracts to prevent oral biofilm on teeth.

## CONFLICT OF INTEREST

The authors declare no conflict of interests.

## Supporting information


**Appendix S1** Supporting information

## Data Availability

Data available in article supplementary material. The data set generated and analyzed in this study is available upon reasonable request from the corresponding author.
