# Supplementary material for: Evaluating hop extract concentrations found in commercial beer to inhibit Streptococcus mutans biofilm formation
Source: J Appl Microbiol. 2022 Jun 5;133(3):1333–40. doi: 10.1111/jam.15632 (PMC9543398; doi:10.1111/jam.15632)
Supplement: Supplementary file 1 — Appendix S1 Supporting information [file JAM-133-1333-s001.docx]

**Supplemental Table 1**

**Mean (Standard Error of the Mean) Values for Each Hop and Dilution Level for Inhibition of Total Absorbance, Biofilm and Planktonic Growth on *S. mutans* UA159**

|  | **Hop** | **1:2** | **1:4** | **1:8** | **1:16** | **1:32** | **1:64** | **1:128** | **1:256** | **Control** |
| --- | --- | --- | --- | --- | --- | --- | --- | --- | --- | --- |
| **Absorbance (Total)** | **A** | 0.108 (0.026) | 0.123 (0.031) | 0.161 (0.033) | 0.223 (0.040) | 0.271 (0.034) | 0.311 (0.036) | 0.338 (0.029) | 0.309 (0.023) | 0.271 (0.029) |
|  | **B** | 0.055 (0.002) | 0.056 (0.003) | 0.093 (0.006) | 0.160 (0.019) | 0.204 (0.014) | 0.259 (0.014) | 0.297 (0.015) | 0.307 (0.016) | 0.344 (0.021) |
|  | **C** | 0.053 (0.002) | 0.059 (0.003) | 0.086 (0.005) | 0.138 (0.005) | 0.149 (0.025) | 0.187 (0.032) | 0.250 (0.034) | 0.223 (0.039) | 0.340 (0.023) |
|  | **D** | 0.049 (0.020) | 0.070 (0.022) | 0.097 (0.016) | 0.140 (0.013) | 0.198 (0.015) | 0.195 (0.022) | 0.245 (0.027) | 0.220 (0.041) | 0.420 (0.046) |
|  | **E** | 0.096 (0.028) | 0.116 (0.035) | 0.170 (0.039) | 0.216 (0.043) | 0.275 (0.041) | 0.327 (0.036) | 0.354 (0.039) | 0.358 (0.037) | 0.357 (0.030) |
|  | **F** | 0.071 (0.029) | 0.098 (0.035) | 0.152 (0.037) | 0.213 (0.040) | 0.288 (0.039) | 0.334 (0.035) | 0.363 (0.037) | 0.379 (0.040) | 0.357 (0.030) |
|  | **G** | 0.079 (0.016) | 0.079 (0.022) | 0.152 (0.053) | 0.133 (0.018) | 0.177 (0.019) | 0.187 (0.022) | 0.219 (0.038) | 0.185 (0.040) | 0.420 (0.046) |
|  | **H** | 0.034 (0.001) | 0.038 (0.001) | 0.047 (0.003) | 0.085 (0.008) | 0.138 (0.014) | 0.170 (0.023) | 0.232 (0.020) | 0.268 (0.026) | 0.340 (0.023) |
|  | **I** | 0.037 (0.004) | 0.043 (0.004) | 0.089 (0.011) | 0.136 (0.012) | 0.201 (0.017) | 0.239 (0.010) | 0.267 (0.010) | 0.305 (0.017) | 0.344 (0.021) |
|  | **J** | 0.096 (0.026) | 0.123 (0.034) | 0.139 (0.033) | 0.206 (0.036) | 0.269 (0.036) | 0.305 (0.030) | 0.324 (0.030) | 0.332 (0.026) | 0.271 (0.029) |
| **Biofilm** | **A** | -0.028 (0.031) | 0.066 (0.034) | 0.164 (0.037) | 0.248 (0.052) | 0.300 (0.061) | 0.455 (0.043) | 0.465 (0.070) | 0.420 (0.063) | 0.415 (0.062) |
|  | **B** | 0.128 (0.038) | 0.230 (0.053) | 0.382 (0.054) | 0.435 (0.061) | 0.587 (0.060) | 0.559 (0.046) | 0.697 (0.029) | 0.743 (0.058) | 0.749 (0.053) |
|  | **C** | 0.072 (0.013) | 0.141 (0.013) | 0.291 (0.029) | 0.388 (0.049) | 0.325 (0.051) | 0.456 (0.064) | 0.566 (0.070) | 0.545 (0.101) | 0.669 (0.060) |
|  | **D** | 0.050 (0.014) | 0.072 (0.012) | 0.273 (0.060) | 0.249 (0.040) | 0.370 (0.052) | 0.448 (0.100) | 0.478 (0.063) | 0.477 (0.084) | 0.537 (0.054) |
|  | **E** | 0.025 (0.016) | 0.078 (0.016) | 0.230 (0.027) | 0.302 (0.018) | 0.425 (0.013) | 0.485 (0.021) | 0.486 (0.020) | 0.468 (0.044) | 0.475 (0.032) |
|  | **F** | 0.015 (0.011) | 0.058 (0.012) | 0.215 (0.021) | 0.300 (0.025) | 0.391 (0.018) | 0.487 (0.012) | 0.496 (0.022) | 0.513 (0.020) | 0.475 (0.032) |
|  | **G** | 0.049 (0.027) | 0.059 (0.007) | 0.191 (0.019) | 0.285 (0.037) | 0.299 (0.047) | 0.415 (0.087) | 0.469 (0.087) | 0.396 (0.083) | 0.537 (0.054) |
|  | **H** | 0.074 (0.019) | 0.094 (0.018) | 0.156 (0.022) | 0.327 (0.043) | 0.348 (0.034) | 0.382 (0.049) | 0.581 (0.056) | 0.593 (0.052) | 0.669 (0.060) |
|  | **I** | 0.163 (0.051) | 0.191 (0.041) | 0.315 (0.063) | 0.498 (0.065) | 0.493 (0.036) | 0.557 (0.050) | 0.517 (0.053) | 0.693 (0.057) | 0.749 (0.053) |
|  | **J** | -0.022 (0.025) | 0.030 (0.020) | 0.131 (0.026) | 0.240 (0.038) | 0.328 (0.044) | 0.377 (0.073) | 0.431 (0.081) | 0.447 (0.083) | 0.415 (0.062) |
| **Planktonic** | **A** | 0.016 (0.003) | 0.007 (0.001) | 0.025 (0.009) | 0.032 (0.009) | 0.042 (0.010) | 0.047 (0.012) | 0.046 (0.011) | 0.041 (0.010) | 0.032 (0.006) |
|  | **B** | 0.013 (0.002) | 0.009 (0.002) | 0.012 (0.001) | 0.028 (0.008) | 0.021 (0.002) | 0.027 (0.004) | 0.021 (0.002) | 0.025 (0.004) | 0.036 (0.003) |
|  | **C** | 0.028 (0.013) | 0.010 (0.002) | 0.013 (0.001) | 0.018 (0.002) | 0.022 (0.003) | 0.025 (0.005) | 0.027 (0.004) | 0.028 (0.006) | 0.070 (0.008) |
|  | **D** | 0.002 (0.001) | 0.006 (0.002) | 0.008 (0.001) | 0.014 (0.002) | 0.021 (0.003) | 0.018 (0.004) | 0.027 (0.003) | 0.022 (0.005) | 0.060 (0.010) |
|  | **E** | 0.021 (0.005) | 0.014 (0.002) | 0.017 (0.005) | 0.024 (0.005) | 0.027 (0.003) | 0.027 (0.003) | 0.033 (0.005) | 0.045 (0.015) | 0.046 (0.005) |
|  | **F** | 0.005 (0.001) | 0.007 (0.002) | 0.041 (0.032) | 0.018 (0.002) | 0.025 (0.002) | 0.031 (0.004) | 0.038 (0.005) | 0.030 (0.005) | 0.046 (0.005) |
|  | **G** | 0.063 (0.050) | 0.007 (0.001) | 0.010 (0.001) | 0.014 (0.002) | 0.022 (0.003) | 0.023 (0.005) | 0.025 (0.004) | 0.024 (0.006) | 0.060 (0.010) |
|  | **H** | 0.010 (0.001) | 0.013 (0.001) | 0.014 (0.001) | 0.015 (0.002) | 0.020 (0.003) | 0.025 (0.004) | 0.032 (0.004) | 0.036 (0.004) | 0.070 (0.008) |
|  | **I** | 0.006 (0.002) | 0.007 (0.002) | 0.017 (0.005) | 0.014 (0.001) | 0.020 (0.002) | 0.040 (0.010) | 0.042 (0.007) | 0.047 (0.012) | 0.036 (0.003) |
|  | **J** | 0.011 (0.001) | 0.008 (0.001) | 0.025 (0.012) | 0.028 (0.008) | 0.036 (0.008) | 0.047 (0.013) | 0.048 (0.013) | 0.086 (0.039) | 0.032 (0.006) |

A-Styrian Celeia (St. Golding); B-Liberty; C-German Hallertau; D-Nugget; E-Czech Saaz; F-Cluster; G-German Northern Brewer; H-Amarillo; I-Cascade; and J-Glacier.

**Supplemental Table 2**

**Rank p-values from One-Way ANOVA Comparing the Hop Dilutions to their Controls for Inhibition of Total Absorbance, Biofilm and Planktonic Growth on *S. mutans* UA159**

|  | **Hop** | **1:2 vs. Control** | **1:4 vs. Control** | **1:8 vs. Control** | **1:16 vs. Control** | **1:32 vs. Control** | **1:64 vs. Control** | **1:128 vs. Control** | **1:256 vs. Control** |
| --- | --- | --- | --- | --- | --- | --- | --- | --- | --- |
| **Absorbance** | **A** | <0.01 | <0.01 | <0.01 | <0.01 | <0.01 | <0.01 | 0.07 | <0.01 |
|  | **B** | <0.01 | <0.01 | <0.01 | <0.01 | <0.01 | <0.01 | 0.23 | 0.60 |
|  | **C** | <0.01 | <0.01 | <0.01 | <0.01 | <0.01 | <0.01 | 0.40 | 0.14 |
|  | **D** | <0.01 | <0.01 | <0.01 | <0.01 | <0.01 | <0.01 | 0.37 | 0.38 |
|  | **E** | <0.01 | <0.01 | <0.01 | <0.01 | <0.01 | 0.09 | 0.98 | 0.78 |
|  | **F** | <0.01 | <0.01 | <0.01 | <0.01 | <0.01 | 0.01 | 0.78 | 0.63 |
|  | **G** | <0.01 | <0.01 | <0.01 | <0.01 | <0.01 | <0.01 | <0.01 | 0.04 |
|  | **H** | <0.01 | <0.01 | <0.01 | <0.01 | <0.01 | <0.01 | 0.01 | 0.09 |
|  | **I** | <0.01 | <0.01 | <0.01 | <0.01 | <0.01 | <0.01 | <0.01 | 0.28 |
|  | **J** | <0.01 | <0.01 | <0.01 | <0.01 | <0.01 | <0.01 | 0.02 | 0.43 |
| **Biofilm** | **A** | <0.01 | <0.01 | <0.01 | <0.01 | 0.05 | 0.93 | 0.25 | 0.16 |
|  | **B** | <0.01 | <0.01 | <0.01 | <0.01 | <0.01 | 0.38 | 0.06 | 0.68 |
|  | **C** | <0.01 | <0.01 | <0.01 | <0.01 | <0.01 | 0.16 | 0.18 | 0.60 |
|  | **D** | <0.01 | <0.01 | <0.01 | <0.01 | <0.01 | <0.01 | 0.35 | 0.09 |
|  | **E** | <0.01 | <0.01 | <0.01 | <0.01 | <0.01 | 0.93 | 0.18 | 0.21 |
|  | **F** | <0.01 | <0.01 | <0.01 | <0.01 | <0.01 | 0.04 | 0.85 | 0.70 |
|  | **G** | <0.01 | <0.01 | <0.01 | <0.01 | <0.01 | <0.01 | 0.01 | <0.01 |
|  | **H** | <0.01 | <0.01 | <0.01 | <0.01 | <0.01 | <0.01 | 0.66 | 0.02 |
|  | **I** | <0.01 | <0.01 | <0.01 | <0.01 | <0.01 | 0.06 | 0.42 | 0.72 |
|  | **J** | <0.01 | <0.01 | <0.01 | <0.01 | 0.03 | 0.94 | 0.22 | 0.08 |
| **Planktonic** | **A** | <0.01 | <0.01 | <0.01 | 0.01 | 0.19 | 0.07 | 0.09 | 0.01 |
|  | **B** | <0.01 | <0.01 | <0.01 | <0.01 | <0.01 | 0.02 | <0.01 | <0.01 |
|  | **C** | <0.01 | <0.01 | <0.01 | <0.01 | <0.01 | <0.01 | <0.01 | <0.01 |
|  | **D** | <0.01 | <0.01 | <0.01 | <0.01 | <0.01 | <0.01 | 0.16 | 0.01 |
|  | **E** | <0.01 | <0.01 | <0.01 | <0.01 | <0.01 | <0.01 | 0.32 | 0.01 |
|  | **F** | <0.01 | <0.01 | <0.01 | <0.01 | <0.01 | <0.01 | 0.45 | <0.01 |
|  | **G** | <0.01 | <0.01 | <0.01 | <0.01 | 0.01 | 0.01 | 0.24 | 0.37 |
|  | **H** | <0.01 | <0.01 | <0.01 | <0.01 | <0.01 | <0.01 | <0.01 | <0.01 |
|  | **I** | <0.01 | <0.01 | <0.01 | <0.01 | <0.01 | <0.01 | 0.07 | 0.31 |
|  | **J** | <0.01 | <0.01 | <0.01 | <0.01 | <0.01 | 0.86 | 0.68 | 0.80 |

A-Styrian Celeia (St. Golding); B-Liberty; C-German Hallertau; D-Nugget; E-Czech Saaz; F-Cluster; G-German Northern Brewer; H-Amarillo; I-Cascade; and J-Glacier.
